# Molecular Backgrounds of ERAP1 Downregulation in Cervical Carcinoma

**DOI:** 10.1155/2015/367837

**Published:** 2015-06-04

**Authors:** Akash M. Mehta, Michelle Osse, Sandra Kolkman-Uljee, Gert Jan Fleuren, Ekaterina S. Jordanova

**Affiliations:** Department of Pathology, Leiden University Medical Center, L1-Q, 2333 ZA Leiden, Netherlands

## Abstract

The antigen processing machinery (APM) plays an important role in immune recognition of virally infected and transformed cells. Defective expression of the APM component ERAP1 is associated with progression and poor clinical outcome in cervical carcinoma. However, the underlying mechanisms of ERAP1 protein downregulation remain to be established. We investigated *ERAP1* mRNA expression levels in 14 patients with established ERAP1 protein downregulation. To further examine the possible pretranscriptional mechanisms of ERAP1 downregulation, *ERAP1* DNA mutation status was analyzed alongside existing data on various single nucleotide polymorphisms. Moreover, loss of heterozygosity at various loci in the *ERAP1* gene was investigated. In cases with ERAP1 protein downregulation, ERAP1 mRNA quantities were found to be significantly lower than in a cohort with normal ERAP1 protein expression (*P* = 0.001). Loss of heterozygosity was demonstrated to occur in up to 50% of tumors with ERAP1 downregulation. Our data indicate that ERAP1 downregulation is associated with loss of heterozygosity. These data provide the first insight into in vivo mechanisms of ERAP1 downregulation in cervical carcinoma.

## 1. Introduction

Cervical carcinoma is the third most common cause of cancer-related death among women worldwide. It is caused by infection of the uterine cervix epithelium by oncogenic types of the human papillomavirus (HPV), followed by viral persistence and progressive malignant transformation, leading to a spectrum of premalignant lesions (cervical intraepithelial neoplasia; CIN) and, ultimately, cervical carcinoma [1]. The antigen processing machinery (APM) and human leukocyte antigen (HLA) class I-mediated peptide presentation are important determinants of the processing and presentation of HPV-derived peptides and are therefore significant factors in recognition and subsequent lysis of cervical carcinoma cells by cytotoxic T lymphocytes [2].

Downregulation of various APM components and of HLA class I has been shown to be associated with detrimental survival among cervical carcinoma patients [3]; in particular, downregulation of ERAP1 (endoplasmic reticulum aminopeptidase associated with antigen presentation 1) has been demonstrated to be an independent predictor of overall and disease-free survival [3]. ERAP1 is responsible for length-specific N-terminal trimming of peptides (derived from intracellular proteins) before presentation by HLA class I molecules [4, 5]. It is therefore hypothesized to be an important determinant of the repertoire of presented peptides, as has been shown in various ERAP1 downregulated mouse models [5–7]. Moreover, genetic variation in the* ERAP1* gene is associated with both increased cervical carcinoma risk and decreased survival among patients [8–10]. These data suggest that ERAP1 is an important factor in tumor immunogenicity and cervical carcinogenesis [11]. In addition, downregulation of ERAP1 protein expression (occurring in approximately 15% of cases) is associated with worse clinical outcome in cervical carcinoma patients [3].

Although several studies have shown that various viral proteins, including the HPV E7 oncoprotein, can interfere with APM-related cellular processes (physically or at the transcriptional level) [12], the mechanisms leading to ERAP1 downregulation in cervical carcinoma remain largely unknown. Recent data suggest that a single nucleotide polymorphism (SNP) in the gene is associated with downregulation of the corresponding protein [9]; however, the existence of a direct causal relation between this SNP and downregulation remains to be established.

To address the issue of ERAP1 downregulation in cervical carcinoma, we have examined* ERAP1* mRNA expression in tumor cells from a panel of cervical carcinoma lesions with known ERAP1 downregulation at the protein level. Subsequently, we performed an evaluation of possible molecular mechanisms for inactivation of the* ERAP1* gene in flow sorted tumor cells from this series of clinical specimens.

## 2. Materials and Methods

### 2.1. Tumor Specimens

From 109 patients with cervical carcinoma who underwent radical hysterectomy with bilateral pelvic lymphadenectomy (by the same surgical team) between 1985 and 1999, formalin-fixed, paraffin-embedded tissue blocks were retrieved from the archives of the Department of Pathology, Leiden University Medical Center, Netherlands. All patients were inhabitants of Netherlands and had not received preoperative radiotherapy or chemotherapy. Mean age was 48.5 years, the youngest patient being 24 years and the oldest 87 years at the time of surgery. The use of clinical material was approved by the institutional review board according to the guidelines of the Dutch Federation of Medical Research Associations; specific patient consent for this study was waived as patients had given general consent for use of operative specimens for research purposes at the time of surgery.

### 2.2. Tumor Dissociation, Staining, Flow Sorting, and DNA Extraction

Formalin-fixed, paraffin-embedded cervical carcinoma tissue blocks (identified by previous immunohistochemical staining of the tissue microarray) were trimmed if necessary to remove normal epithelium and CIN. The remaining tumor tissue was dissociated, stained for keratin, vimentin, and DNA as previously described [13]. The samples were analysed using a FACS Calibur (BD Biosciences, San Jose, CA). The vimentin-negative keratin-positive (V−K+) fraction, which represents the epithelial cell (tumor cell) subpopulation, and vimentin-positive keratin-negative (V+K−) fraction, consisting of normal (stromal) cells, were flow sorted using a FACS Vantage (BD Biosciences, San Jose, CA). As previously described, this method yields a high range of purity of the different sorted factions [13]. Sorted cells were resuspended in isolation buffer (0.3 mg/mL proteinase K in 10 mM Tris-HCl (pH 8.3), 1 mM EDTA, and 0.5% Tween 20) at a concentration of 1000 cells/*μ*L. After overnight incubation at 56°C and 5 min inactivation at 100°C, DNA samples were stored at −20°C until further analysis [14].

### 2.3. DNA Mutation Analysis

DNA amplification and sequencing were performed on flow sorted tumor and normal fractions as previously described with CAACCTCGCAGCTTCCCCGG (nucleotide position 54 → 73 of GenBank accession number NM_016442) and GGCCTAGCTCCCCCAGGACC (nucleotide position 276 → 257) as forward and reverse primers, respectively [15]. All sequences were visually analyzed with Mutation Surveyor DNA variant analysis software (Version 2.61; Softgenetics, State College, PA).

### 2.4. RNA Isolation and Quantitative Real-Time PCR (QRT-PCR)

RNA was isolated from macrodissected formalin-fixed, paraffin-embedded material and cDNA synthesis, qPCR, and analysis were performed as described previously [16]. Expression of* ERAP1*-specific mRNA was determined by QRT-PCR in an iCycler iQ (Bio-Rad Laboratories Inc., Hercules, CA) using CATCGGTTGGATGGATAAGAA (nucleotide position 3101 → 3121 of GenBank accession number NM_016442) and CATCCTGTTGCGTCAGCTT (nucleotide position 3193 → 3175) as forward and reverse primers, respectively. Besides* ERAP1*, expression of the housekeeping gene beta-actin (*ACTB*) was also measured with primer sequences as described previously [17]. Expression of these genes was measured using the Sybr Green method (Eurogentec, Seraing, Belgium) according to the manufacturer's instructions. For each measured gene, the required calibration curve was determined using a serial dilution of pooled cDNA from established cervical cancer cell lines (SIHA, Caski, C33, and Hela). Each measurement was performed in duplicate.

For the analysis,* ERAP1* mRNA expression levels were calculated relative to the ACTB housekeeping gene for both ERAP1 downregulated cases and control specimens. Mean* ERAP1* mRNA expression levels of both groups were compared using the independent samples *t*-test.

### 2.5. Microsatellite and Allelic Status Analysis

To investigate loss or retention of heterozygosity (LOH and ROH, resp.) of the* ERAP1* gene locus on chromosome 5q15, in DNA isolated from flow sorted samples of the 16 ERAP1 downregulated cases, 4 microsatellite markers (D5S433, D5S1463, D5S107, and D5S2094) spanning the genomic region from 5q21 to 5q14 were amplified using 6-carboxy-2′,4,4′,5′,7,7′-hexachlorofluorescein- (HEX-) labeled primer pairs (Genome Database, http://www.gdb.org/). As no suitable microsatellite marker could be identified in the* ERAP1* gene itself, 5 previously described single nucleotide polymorphisms (SNPs) (codons 56, 127, 276, 528, and 730 of the ERAP1 gene) were investigated using TaqMan SNP Genotyping Assays (Applied Biosystems, Inc., Foster City, CA) as described previously [8]. PCR, electrophoresis, and analysis were performed as described previously [18].

Since flow sorting yields highly pure stromal and tumor cell fractions, the threshold for complete LOH in analysis of the microsatellite markers was set high [19]. Comparing normal and tumor DNA, a reduction of at least 80% of one allele was defined as complete LOH, corresponding to an allelic imbalance factor (AIF) of ≥5. Retention of heterozygosity was defined as less than 30% reduction of one allele (AIF ≤ 1.4). The range between 1.5 and 5 was defined as allelic imbalance (AI). Haplotype loss was defined as loss of at least three markers.

## 3. Results

### 3.1. ERAP1 Protein and mRNA Expression

Results of immunohistochemical staining of the tissue microarray using an anti-ERAP1 antibody have been described previously [3]. In short, 16 cases (15%) showed partial loss of ERAP1 expression while normal expression was observed in the remaining cases. Moreover, ERAP1 downregulation was shown to be an independent prognostic parameter for shorter overall and disease-free survival (*P* = 0.037 and *P* = 0.049, resp.).

**Figure 1:**
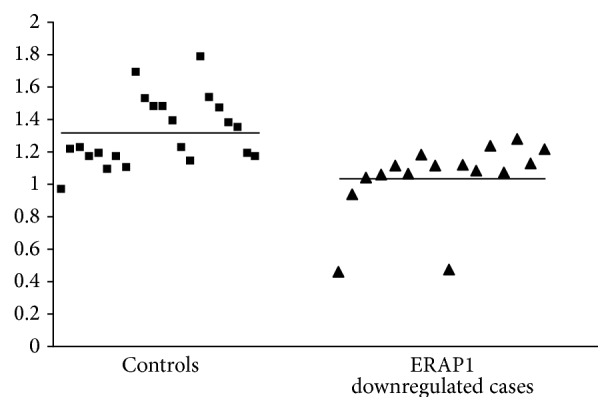
ERAP1 mRNA expression levels relative to the ACTB housekeeping gene in control patients with normal ERAP1 protein expression and cases with ERAP1 protein downregulation. The horizontal lines indicate the mean expression levels in the two groups.

As it was unclear whether downregulation of ERAP1 was influenced by factors at a pre- or posttranscriptional level, mRNA was isolated from 14 cases with ERAP1 downregulation and from 22 randomly selected cases with normal ERAP1 expression levels. Distribution of the* ERAP1* mRNA expression levels in both groups is demonstrated in Figure 1. A 21% downregulation of* ERAP1* mRNA expression was found in the group with ERAP1 protein downregulation as compared to the group with normal protein expression (*P* = 0.001). Even when the cases with the lowest mRNA expression levels were excluded from analysis, the observed difference was still significant (*P* = 0.003).

To investigate the underlying mechanisms of ERAP1 protein and mRNA downregulation, the role of various molecular factors was tested.

### 3.2. Genotype Analysis

The results of analysis of various* ERAP1* SNPs in relation to protein expression have been described previously [9]. Recently, a mutation in codon 349 (nucleotide position 1045; c.1045A>G) of the* ERAP1* gene was reported in melanoma cell lines [20]; to investigate association of this mutation with protein expression, mutation analysis on DNA from pure tumor cell fractions (obtained by flow sorting) of the 16 ERAP1 downregulated cases was performed. The* ERAP1*-349 mutation was not found in any of the specimens with ERAP1 protein downregulation (data not shown).

### 3.3. LOH Analysis

**Figure 2:**
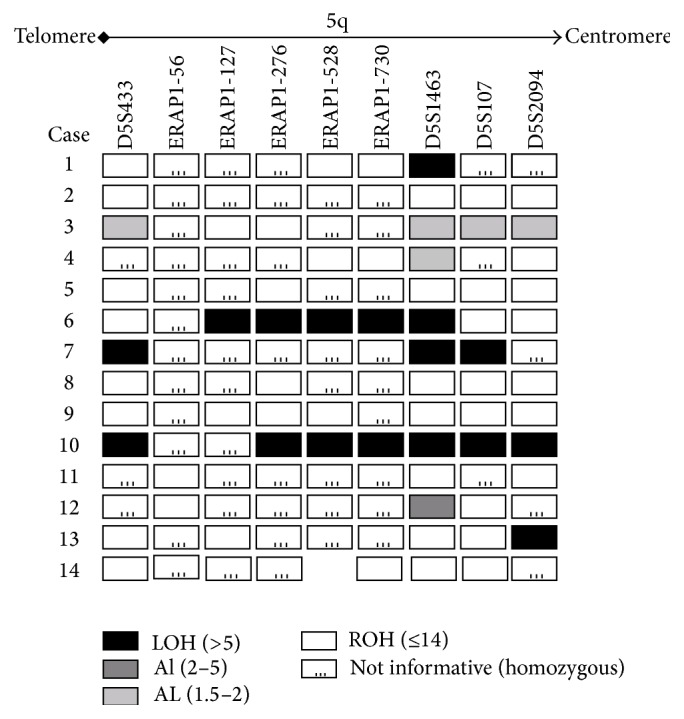
Patterns of microsatellite instability in 14 patients with ERAP1 downregulation.

As partial or complete LOH has been shown to be an important factor in various human carcinomas, the role of allelic instability in ERAP1 downregulation was assessed. Of the 16 specimens with ERAP1 downregulation, 14 were successfully analysed for LOH (Figure 2). The four microsatellite markers D5S433, D5S1463, D5S107, and D5S2094 exhibited either LOH or allelic imbalance in 21%–50% of cases, with complete LOH occurring in 14%–29% of cases. Overall, haplotype loss occurred in 21% of cases.

## 4. Discussion

A key pathway in antitumor immunity is the presentation of tumor derived peptides by HLA class I molecules to cytotoxic T lymphocytes. The formation and intracellular transport of these peptides are performed by the various components of the antigen processing machinery (APM). One of these components, ERAP1, has previously been shown to be an important determinant of the repertoire of HLA class I-presented peptides [4, 6, 7, 21, 22]. Furthermore, genetic variation in the* ERAP1* gene has been shown to be significantly associated with risk of developing cervical carcinoma and with prognosis of cervical carcinoma patients [8–10]; specifically, a SNP at the* ERAP1-127* locus was shown to be an independent predictor of both cervical carcinoma risk and cervical carcinoma-associated survival, possibly due to its location in the peptidase M1 domain of the ERAP protein [9]. Importantly, we have previously shown that ERAP1 downregulation is significantly associated with decreased survival in cervical carcinoma [3]. However, to date the mechanisms leading to ERAP1 downregulation in this tumor type have not been elucidated. Although a previous study has investigated the underlying mechanisms of ERAP1 downregulation in melanoma cell lines [20], no investigations in clinical specimens have been performed to date. The present study therefore is the first description of such mechanisms in clinical specimens.

Firstly, we investigated whether the processes leading to protein downregulation occur at a pre- or posttranscriptional level by studying* ERAP1* mRNA levels in patients with normal and decreased ERAP1 expression. mRNA downregulation was found to be significantly associated with decreased protein expression, which indicates that the factors influencing ERAP1 expression occur at a pretranscriptional level.

The possible pretranscriptional mechanisms of protein downregulation can be both genetic and epigenetic. As previous reports indicate that DNA methylation status is not a contributing factor in ERAP1 downregulation [20, 23], we did not investigate* ERAP1* gene methylation in the present study.

At the genetic level, both single nucleotide polymorphisms (SNPs) and mutations can lead to altered expression. In our previous report, 5 SNPs (*ERAP1*-56,* ERAP1*-127,* ERAP1*-276,* ERAP1*-528, and* ERAP1*-730) in the* ERAP1* gene were genotyped and investigated for association with ERAP1 protein downregulation; genetic variation at ERAP1-127 was found to be significantly associated with ERAP1 protein expression (*P* < 0.001) [9]. Until recently, no* ERAP1* mutations had been studied in human tumors. However, a recent study identified a novel* ERAP1* mutation in human melanoma cell lines [20]. This mutation could not be found in any of the ERAP1 cases showing low ERAP1 expression, nor in any patients with normal ERAP1 expression.

In addition to SNPs and mutations, loss of heterozygosity has been shown to be an important genetic factor in human carcinomas; in the present study, complete and partial LOH and haplotype loss were found to occur in a considerable proportion of cases with ERAP1 downregulation (up to 50%).

LOH and haplotype loss are relatively frequently occurring phenomena in various human carcinomas and have been found to be related to prognosis in colon carcinoma [24]. Our findings suggest that these phenomena occur frequently in cervical carcinoma with ERAP1 downregulation.

In conjunction with our previous reports on the association of molecular changes with clinical aspects, the present study is part of an ongoing effort to elucidate the effects of interaction between exogenous and endogenous factors in the etiology, pathogenesis, and prognosis of cervical cancer. This field, recently termed molecular pathological epidemiology (MPE), has made considerable progress in colorectal carcinoma [24–29]; we believe that our study is the start of the first MPE-based investigative analysis in cervical carcinoma. Our finding that different genetic factors play a role in ERAP1 downregulated cervical carcinogenesis suggests that various exogenous factors interact with host genotypes to define susceptibility to malignant transformation [30]. Studies substratifying the molecular changes reported here by factors such as HPV type and lifestyle are necessary to further elucidate the mechanisms ultimately responsible for development and progression of cervical carcinoma.

In conclusion, here we present the results of the first investigation of in vivo mechanisms of ERAP1 downregulation in cervical carcinoma. Taken together, the results of this study indicate that ERAP1 protein downregulation occurs at a pretranscriptional level and is associated with both genetic variation in SNPs and loss of heterozygosity. As ERAP1 downregulation has been shown to be a significant predictor of decreased survival in cervical carcinoma, insight into the possible underlying mechanisms of this downregulation may yield novel targets for the designs of antitumor immunotherapies.

## References

[1] Stern P. L. (1996). Immunity to human papillomavirus-associated cervical neoplasia. *Advances in Cancer Research*.

[2] Heemels M.-T., Ploegh H. (1995). Generation, translocation, and presentation of MHC class I-restricted peptides. *Annual Review of Biochemistry*.

[3] Mehta A. M., Jordanova E. S., Kenter G. G., Ferrone S., Fleuren G.-J. (2008). Association of antigen processing machinery and HLA class I defects with clinicopathological outcome in cervical carcinoma. *Cancer Immunology, Immunotherapy*.

[4] York I. A., Chang S.-C., Saric T. (2002). The ER aminopeptidase ERAP1 enhances or limits antigen presentation by trimming epitopes to 8-9 residues. *Nature Immunology*.

[5] York I. A., Brehm M. A., Zendzian S., Towne C. F., Rock K. L. (2006). Endoplasmic reticulum aminopeptidase 1 (ERAP1) trims MHC class I-presented peptides in vivo and plays an important role in immunodominance. *Proceedings of the National Academy of Sciences of the United States of America*.

[6] Hammer G. E., Gonzalez F., Champsaur M., Cado D., Shastri N. (2006). The aminopeptidase ERAAP shapes the peptide repertoire displayed by major histocompatibility complex class I molecules. *Nature Immunology*.

[7] Yan J., Parekh V. V., Mendez-Fernandez Y. (2006). *In vivo* role of ER-associated peptidase activity in tailoring peptides for presentation by MHC class Ia and class Ib molecules. *The Journal of Experimental Medicine*.

[8] Mehta A. M., Jordanova E. S., van Wezel T. (2007). Genetic variation of antigen processing machinery components and association with cervical carcinoma. *Genes Chromosomes and Cancer*.

[9] Mehta A. M., Jordanova E. S., Corver W. E. (2009). Single nucleotide polymorphisms in antigen processing machinery component ERAP1 significantly associate with clinical outcome in cervical carcinoma. *Genes, Chromosomes and Cancer*.

[10] Mehta A. M., Spaans V. M., Mahendra N. B. (2015). Differences in genetic variation in antigen-processing machinery components and association with cervical carcinoma risk in two Indonesian populations. *Immunogenetics*.

[11] Stratikos E., Stamogiannos A., Zervoudi E., Fruci D. (2014). A role for naturally occurring alleles of endoplasmic reticulum aminopeptidases in tumor immunity and cancer pre-disposition. *Frontiers in Oncology*.

[12] Seliger B., Ritz U., Ferrone S. (2006). Molecular mechanisms of HLA class I antigen abnormalities following viral infection and transformation. *International Journal of Cancer*.

[13] Corver W. E., ter Haar N. T., Dreef E. J. (2005). High-resolution multi-parameter DNA flow cytometry enables detection of tumour and stromal cell subpopulations in paraffin-embedded tissues. *Journal of Pathology*.

[14] Abeln E. C. A., Corver W. E., Kuipers-Dijkshoorn N. J., Fleuren G.-J., Cornelisse C. J. (1994). Molecular genetic analysis of ovarian tumour cells: improved detection of loss of heterozygosity. *British Journal of Cancer*.

[15] van Eijk R., van Puijenbroek M., Chhatta A. R. (2010). Sensitive and specific KRAS somatic mutation analysis on whole-genome amplified DNA from archival tissues. *Journal of Molecular Diagnostics*.

[16] de Boer M. A., Jordanova E. S., Kenter G. G. (2007). High human papillomavirus oncogene mRNA expression and not viral DNA load is associated with poor prognosis in cervical cancer patients. *Clinical Cancer Research*.

[17] Andersen C. L., Jensen J. L., Ørntoft T. F. (2004). Normalization of real-time quantitative reverse transcription-PCR data: a model-based variance estimation approach to identify genes suited for normalization, applied to bladder and colon cancer data sets. *Cancer Research*.

[18] Haven C. J., van Puijenbroek M., Karperien M., Fleuren G.-J., Morreau H. (2004). Differential expression of the calcium sensing receptor and combined loss of chromosomes Iq and IIq in parathyroid carcinoma. *The Journal of Pathology*.

[19] Devilee P., Cleton-Jansen A.-M., Cornelisse C. J. (2001). Ever since Knudson. *Trends in Genetics*.

[20] Kamphausen E., Kellert C., Abbas T. (2010). Distinct molecular mechanisms leading to deficient expression of ER-resident aminopeptidases in melanoma. *Cancer Immunology, Immunotherapy*.

[21] Grommé M., Neefjes J. (2002). Antigen degradation or presentation by MHC class I molecules via classical and non-classical pathways. *Molecular Immunology*.

[22] Van Kaer L. (2002). Major histocompatibility complex class I-restricted antigen processing and presentation. *Tissue Antigens*.

[23] Hasim A., Abudula M., Aimiduo R. (2012). Post-transcriptional and epigenetic regulation of antigen processing machinery (APM) components and HLA-I in cervical cancers from Uighur women. *PLoS ONE*.

[24] Ogino S., Nosho K., Irahara N. (2009). Prognostic significance and molecular associations of 18q loss of heterozygosity: a cohort study of microsatellite stable colorectal cancers. *Journal of Clinical Oncology*.

[25] Campbell P. T., Jacobs E. T., Ulrich C. M. (2010). Case-control study of overweight, obesity, and colorectal cancer risk, overall and by tumor microsatellite instability status. *Journal of the National Cancer Institute*.

[26] Ogino S., Chan A. T., Fuchs C. S., Giovannucci E. (2011). Molecular pathological epidemiology of colorectal neoplasia: an emerging transdisciplinary and interdisciplinary field. *Gut*.

[27] Song M., Nishihara R., Wang M. (2015). Plasma 25-hydroxyvitamin D and colorectal cancer risk according to tumour immunity status. *Gut*.

[28] Inamura K., Yamauchi M., Nishihara R. (2014). Tumor LINE-1 methylation level and microsatellite instability in relation to colorectal cancer prognosis. *Journal of the National Cancer Institute*.

[29] Inamura K., Yamauchi M., Nishihara R. (2015). Prognostic significance and molecular features of signet-ring cell and mucinous components in colorectal carcinoma. *Annals of Surgical Oncology*.

[30] Ogino S., Lochhead P., Chan A. T. (2013). Molecular pathological epidemiology of epigenetics: emerging integrative science to analyze environment, host, and disease. *Modern Pathology*.

